# ORGANIZATION OF WORK AND DECISION-MAKING OF PROFESSIONALS WORKING IN NURSING HOMES IN CATALONIA (SPAIN)

**DOI:** 10.1093/geroni/igz038.2573

**Published:** 2019-11-08

**Authors:** Montserrat Gea-Sánchez, Alvaro Alconada-Romero, Roland Pastells-Peiró, Filip Bellon, Lourdes Teres-Vidal, Joan Blanco-Blanco, and Katherine McGilton

**Affiliations:** 1 Faculty of Nursing and Physiotherapy, University of Lleida, Lleida, Spain; 2 Col legi Oficial d’infermeres i infermers de Lleida, Lleida, Spain; 3 KITE, Toronto Rehab, University Health Network, Toronto, Ontario, Canada

## Abstract

The long term care environment demands specific requirements of the staff, namely that they provide a holistic approach to care. Providing holistic care leads to complex decision-making processes which go beyond just finding a solution to a specific health problem. It requires that staff are able to respond to the diverse needs expressed by the residents and which, in many cases, are only identified through the relationship that professionals have with them. However, this relationship that staff establishes with the resident often leads to burden for these professionals. The researchers sought to identify characteristics of the nursing homes that lead to positive outcomes for staff. The study involves collecting questionnaires (n=132) and conducting semi structured interviews (n=35) in 9 Catalan nursing homes with number of staff, utilizing a quantitative questionnaire and semi-structured interviews. Practices in organisations that led to positive outcomes for staff included coordinated care that includes processes of support for staff, effective communication and decision making practices, clear responsibilities for staff, and utilization of care plans. Effective long term care practices can favour both patient care and professional practice in residences for elderly.

